# Measurement of Physician-Patient Communication—A Systematic Review

**DOI:** 10.1371/journal.pone.0112637

**Published:** 2014-12-22

**Authors:** Jördis M. Zill, Eva Christalle, Evamaria Müller, Martin Härter, Jörg Dirmaier, Isabelle Scholl

**Affiliations:** Department of Medical Psychology, University Medical Center Hamburg-Eppendorf, Hamburg, Germany; University of St Andrews, United Kingdom

## Abstract

**Background:**

Effective communication with health care providers has been found as relevant for physical and psychological health outcomes as well as the patients' adherence. However, the validity of the findings depends on the quality of the applied measures. This study aimed to provide an overview of measures of physician-patient communication and to evaluate the methodological quality of psychometric studies and the quality of psychometric properties of the identified measures.

**Methods:**

A systematic review was performed to identify psychometrically tested instruments which measure physician-patient communication. The search strategy included three databases (EMBASE, PsycINFO, PubMed), reference and citation tracking and personal knowledge. Studies that report the psychometric properties of physician-patient communication measures were included. Two independent raters assessed the methodological quality of the selected studies with the COSMIN (COnsensus based Standards for the selection of health status Measurement INtruments) checklist. The quality of psychometric properties was evaluated with the quality criteria of Terwee and colleagues.

**Results:**

Data of 25 studies on 20 measures of physician-patient communication were extracted, mainly from primary care samples in Europe and the USA. Included studies reported a median of 3 out of the nine COSMIN criteria. Scores for internal consistency and content validity were mainly fair or poor. Reliability and structural validity were rated mainly of fair quality. Hypothesis testing scored mostly poor. The quality of psychometric properties of measures evaluated with Terwee et al.'s criteria was rated mainly intermediate or positive.

**Discussion:**

This systematic review identified a number of measures of physician-patient communication. However, further psychometric evaluation of the measures is strongly recommended. The application of quality criteria like the COSMIN checklist could improve the methodological quality of psychometric property studies as well as the comparability of the studies' results.

## Introduction

Over the past decades, patient-centeredness has been an internationally discussed topic in all health care associated fields [1]. Effective and competent communication of physicians with their patients constitutes one of the core dimensions of patient-centeredness [2], [3]. Moreover, communication skills are one of the most relevant components of physicians' overall social competencies and have been identified as one of the six competencies by the Accreditation Council on Graduate Medical Education (ACGME) required for the effective practice of medicine [4], [5].

Although there is no overall consensus on the operational definition of physician-patient communication [3], Street et al. [6] pointed out that core functions of patient-centered communication are the exchange of information, supporting patients' self-management, the management of uncertainty and emotions, decision making and enhancing the physician-patient relationship. Studies have shown that good physician-patient communication skills are associated with patient health outcomes. E.g. Zolnierek et al. [7] found in a meta-analysis that physician-patient communication is significantly positively correlated with patient adherence. They also reported significant improvements of the patients' adherence when physicians received communication training. Other studies found a positive relationship of physician-patient communication and patient satisfaction [8]–[10], and physical health outcomes [2]. Further studies emphasize that effects of enhanced physician-patient-communication on health outcomes, are mainly indirect [3], [6], [11]. Street et al. [6] provided a model that suggests how communication could influence patient health outcomes via direct and indirect pathways. They suggest that proximal outcomes linked to physician-patient communication are, amongst others, patients' trust in the physician, understanding, motivation, involvement and rapport. These outcomes affect intermediate outcomes such as access to care, self-care skills or commitment to treatment, which in turn affect emotional well-being, vitality and health.

Since the importance of physician-patient communication has been widely recognized, a considerable number of instruments that measure physicians' communication skills have been developed. In their review from 1995 Ong et al. [12] addressed different topics related to physician-patient communication. They included a chapter on the different purposes of medical communication, reviewed specific communication behaviors and their influence on patient outcomes, and did an analysis on physician-patient communication. For the latter they presented a brief overview of different measures of physician-patient communication. A further comprehensive review provided by Boon and Stewart [13] compared measures published between 1986 and 1996 which measure physician-patient interaction. They found 44 instruments that were reviewed for reliability and validity. Most instruments were reliable and were designed for teaching and medical education. An up-to-date comparison and evaluation of existing instruments based on clearly defined quality criteria is missing so far, but is necessary to a) choose the most appropriate instrument for a specific research purpose b) facilitate the comparison and appraisal of different intervention studies and c) clarify further research needs, e.g. (re-) development of measurement instruments. Hence, this study seeks to 1) provide a systematic overview of generic measures on physician-patient communication, 2) evaluate the quality of design, methods and reporting of studies that present psychometric properties of measures, and 3) determine the quality of the psychometric properties of the identified measures.

## Methods

The systematic review was registered in the International prospective register of systematic reviews PROSPERO (registration code: CRD42013005687).

### Eligibility criteria

Peer-reviewed studies, published in English or German, were retrieved. We included studies, which tested psychometric properties (e.g. validity, reliability) of instruments that measure the construct physician-patient communication. We adopted a broad definition of communication comprising verbal or non-verbal behavior, a set of communication, interaction or interpersonal skills. We included studies on communication between physicians and adult patients (≥18 years). We excluded studies that only reported communication in a subscale of a broader construct and studies that were limited to the medical education setting. Only generic instruments (i.e. applicable to a broad range of health conditions, groups of patients, and settings) were included for the reason that we found specific measures (e.g. measuring only end-of-life care) were less comparable to each other than to generic measures. The applied inclusion and exclusion criteria are displayed in Table 1.

**Table 1:** Inclusion and Exclusion criteria.

Inclusion criteria	
(**1**)	The full text is accessible
(**2**)	The language of the publication is English or German
(**3**)	The article is published in a peer-reviewed journal
(**4**)	The aim of study is to test psychometric properties of an instrument
(**5**)	The measured construct is communication*
(**6**)	The target group is adult patients
(**7**)	The communication partners are patient and physician

Exclusion criteria	
(**1**)	The main aim of the study is to assess communication skills within medical education setting
(**2**)	Communication is only a subscale of a broader measure
(**3**)	The instrument is condition-specific, specialty-specific, applicable to a subgroup of patients with specific demographic characteristics only (i.e. NOT generic)

*communication (skill); interaction; verbal behavior; non-verbal behavior; interpersonal skill; consultation (skill).

### Search strategy

We searched the databases PubMed, PsycINFO and EMBASE including all articles from their inception to August 15, 2013. For each data base a specific search strategy was developed based on a combination of Medical Subject Headings (MeSH) and free text terms in five domains: (i) patient (ii) physician, (iii) communication, (iv) measurement and (v) psychometrics (see S1 File). Furthermore, we used the PubMed search filter for finding studies on psychometric properties of measures developed by Terwee et al. [14]. This filter was developed by a multidisciplinary team of experts in the field of health status measurement instruments, also known as the COSMIN group (www.cosmin.nl) to facilitate the selection of studies on measurement properties of measurement instruments. We also conducted a secondary search, tracking all reference lists and citations of the included full-texts for further studies of potential relevance and included articles of the authors' personal knowledge.

### Study selection

For an initial screening, all search results were imported into a reference management software (Endnote) and duplicates were removed. First, titles and abstracts were assessed to exclude clearly irrelevant records. Second, the remaining full texts were assessed for eligibility. All steps were performed independently by two team members (EC and JZ or IS or JD). The two members decided upon inclusion. Disagreements between reviewers were resolved in discussion with a third team member (JZ or IS or JD). The reviewers were not blinded to authors, date and journal of publication.

### Data extraction and quality assessment

Three reviewers (EC, JZ and EM) extracted data of the included studies on measures of physician- patient communication by using data extraction sheets. To reduce any bias that may occur with the assessment of one reviewer only, one study was independently assessed triple by EC, JZ and EM. As recommended by Mokkink et al. [15], we did a self-training to ensure all reviewers apply the COSMIN checklist (see section 2.4.2) and Terwee et al.'s criteria (see section 2.4.3) correctly. For another five studies, independent double assessment was performed (either JZ and EM or EC and EM). Initial ambiguities in the rating procedure were discussed between the reviewers and within the research team. After this set of five studies, no further questions occurred and the data extraction and quality rating was performed by one reviewer (either JZ or EC). We sought information about (1) descriptive data of measures and studies (2) quality of design, methods and reporting and (3) quality of psychometric properties of the included studies.

### Descriptive data

The following descriptive data was extracted for the measures: name of the instrument, authors, year, language, perspective (e.g. patient- or physician-reported outcome or observer rating or coding), dimensions, number of items and response scale. Furthermore, we extracted study characteristics (e.g. setting, sample, country).

### Assessment of the methodological quality

The Center for Reviews and Dissemination and the Preferred Reporting Items for Systematic Reviews and Meta-Analyses recommends using checklists for the appraisal of study quality (http://www.prisma-statement.org/). We undertook two assessments of quality, one for the methodological quality of the included studies and one that describes the psychometric quality of the included studies. For the assessment of the methodological quality of the included measures, the COnsensus-based Standards for the selection of health Measurement INstruments (COSMIN) checklist, [16]–[18] was applied. The COSMIN checklist was developed in an international Delphi study that sought consensus on definitions and assessments of measurement properties [17]. For systematic reviews the application of the four-point rating scale has been found as appropriate assessment method [18]. The COSMIN checklist consists of twelve boxes. Nine of these boxes refer to methodological standards for studies on measurement properties: A) internal consistency, B) reliability, C) measurement error, D) content validity, E) structural validity, F) hypotheses testing, G) cross-cultural validity, H) criterion validity, I) responsiveness. Box J) contains two standards for the interpretability of patient-reported outcomes. Furthermore, the COSMIN checklist provides evaluation standards for articles that use the Item-Response-Theory (IRT box) and generalizability of the results (Generalizability box). Each of the boxes A) to I) and the IRT box consist of several items concerning design requirements and statistical analyses. The items can be scored on the four-point rating scale representing options for poor (0), fair (+), good (++) or excellent (+++) quality. The overall score of the quality of each psychometric property is defined as the lowest score of any item within the box, following the “worst score counts” method. Data extraction and evaluation was performed for all COSMIN boxes, but we limited the description to the results of the 4-point scale ratings psychometric property boxes A) to I), since the Generalizability box and the Interpretability (box J) do not add much information to our extraction of descriptive data of the studies. On the COSMIN website (www.cosmin.nl) the authors point out that the checklist mainly focuses on standards for studies that examine psychometric properties of Health-Related Patient-Reported Outcomes (HR-PROs). In this study, we included patient- and self-reported measures as well as observer based measures on physician-patient communication. Nevertheless, prior studies applied the COSMIN criteria to a range of measures [19], [20]. For the instruments that use observer codings or ratings, the items of the COSMIN checklist are not always applicable (e.g. the design requirement on how to handle missing items is not applicable for observer coding systems). Those items of the checklist for the observer tools were coded as “not applicable” (n/a).

### Quality rating of psychometric properties

In order to evaluate and compare the included studies for the quality rating of psychometric properties the criteria developed by Terwee et al. [21] were applied. The criteria refer to the following psychometric properties: content validity, internal consistency, criterion validity, construct validity, reproducibility (agreement and reliability), responsiveness, floor and ceiling effects and interpretability. All properties can be evaluated by one item as either positive (+), intermediate (?), negative (-) or no information available (0). This list of criteria has been applied successfully in prior reviews [22].

### Data analysis and synthesis of results

The key characteristics of the studies and the assessment of the methodological quality and the quality rating of psychometric properties were combined in a narrative summary. For the results of the methodological quality assessment, the median of the number of COSMIN criteria reported in the studies is presented. Furthermore, an overview of the results is displayed in two tables.

## Results

### Literature search and study selection

**Figure 1:**
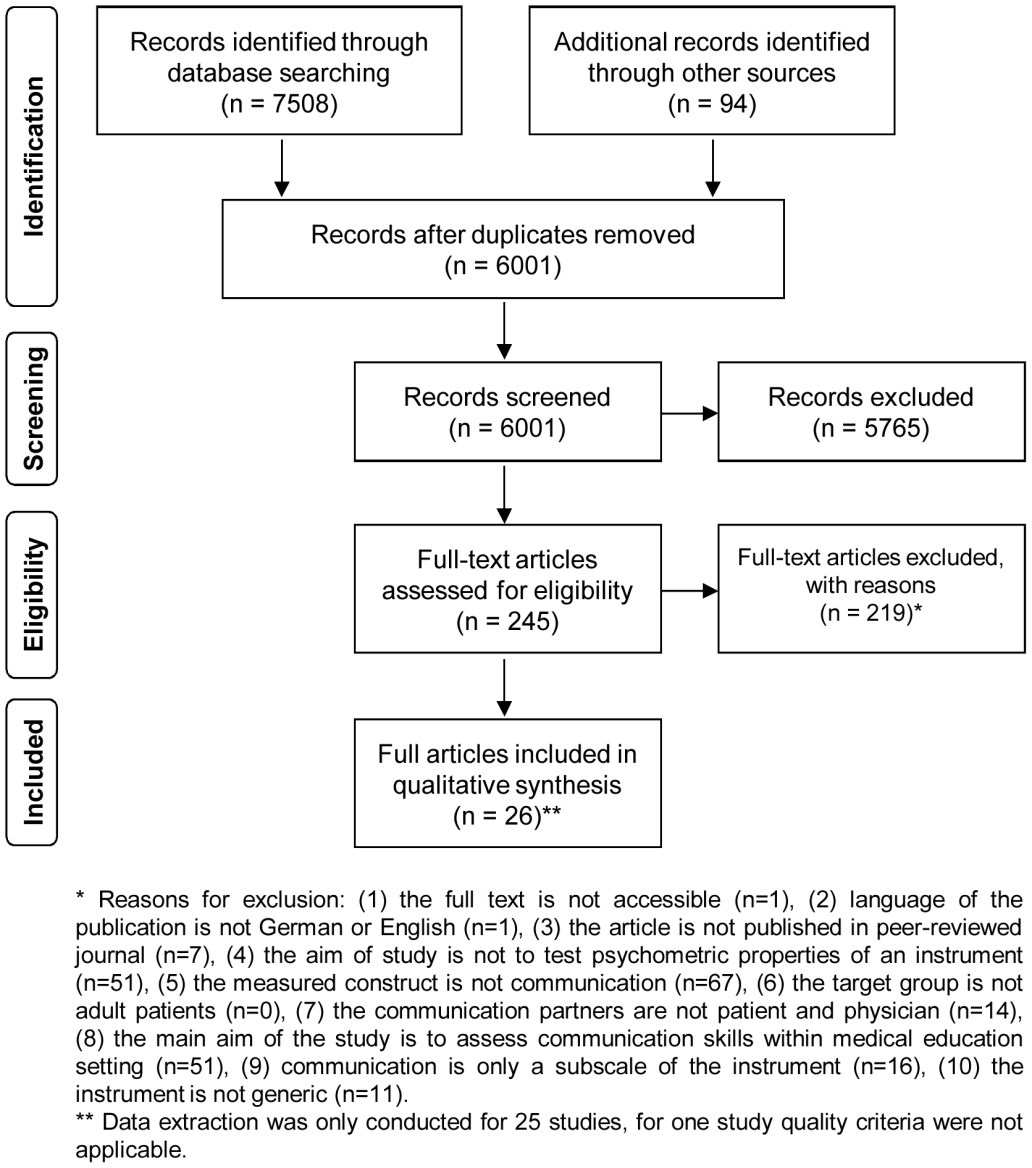
PRISMA flow chart of study selection.

Electronic searches identified 7508 records. The secondary search yielded another 94 records, 92 studies were identified from citation and reference tracking and two studies by the authors' personal knowledge. Duplicates were removed and of the 6001 remaining records, 5765 records were excluded based on title- and abstract screening. The full-texts of 245 records were assessed for eligibility. 219 records did not fulfill the inclusion criteria (see Table 1) and were excluded. This led to the inclusion of 26 studies. The main reasons for exclusion were that the measured construct was not communication (N = 67) or that the aim of study was not to test psychometric properties of an instrument (N = 51) or to measure communication skills within a medical education setting (N = 51). The study selection procedure and reasons for exclusion are displayed in Fig. 1.

The initial studies on the development of the following three instruments [23]–[25] could not be included in this review. For the Classification System of Byrne and Long and the Roter Interaction Analysis System (RIAS) [26], no study on the original development was published in a peer reviewed journal and the publication on the original development study of the VR-MICS was only available in Italian [27]. For three studies, we only extracted one part of the study since these articles described more than only a physicians' version of the measure [28]–[30]. For one study [31], no data extraction was conducted for the reason that we found the structure of the study not transparent and neither COSMIN nor the criteria of Terwee et al. could be applied. Therefore, data on methodological quality and quality of psychometric properties was extracted for 25 studies only.

### Characteristics of included studies

More than half of the studies were conducted in Europe [23]–[25], [30], [32]–[42], seven in the USA [28], [29], [43]–[47], one in Canada [48], one in Japan [49] and one in Kenya [50]. Study settings were mostly outpatient practices, but a few were conducted in (outpatient) departments of hospitals or medical care centers. Sixteen studies were initial studies on the psychometric properties of an instrument [28]–[30], [32]–[34], [39], [40], [42]–[49], eight studies conducted further examination of psychometric properties of a previously developed instrument [23]–[25], [35]–[38], [41], [50]. Characteristics of the included studies are displayed in Table 2.

**Table 2:** Descriptive data of the included studies.

Measure	Authors (Year)	Setting*	Study Sample*	Country
**PPCP**	Shapiro et al. (1981)	family medicine	p: n = 61, 18% m; ph: n = 10, 100% m; audiotapes	USA
**CSBL**	Buijs et al. (1984)	GP's from the Netherlands Institute of General Practitioners	ph: n = 6; 36 video-consultations	Netherlands
**RIAS**	Ong et al. (1998)	1 & 2: cancer patients with their gynecologist/oncologist/urologist	1: p: n = 25, 12% m, mean age 55 ys (r: 28–83); ph: n = 6, 80% m 2: p: n = 60; 25% m, mean age 54 ys (SD 17.9); ph: n = 8, 75% m	Netherlands
		3: three general practices	3: p: n = 329, 36.6% m; mean age 49 ys (SD 16.6); ph: n = 15, 86.7% m/patients: n = 103, 34.9% m, mean age 58 ys (SD 14.9); ph: n = 17, 100% m/p: n = 65, 100% f, mean age 36 ys (SD 13.4); ph: n = 17, 35.2% m	
**MCCS**	Cegala et al. (1998)	general practices	p: n = 52, 35% m, mean age 49 ys (r: 19–89); ph: n = 65, 75% m, mean age 45 ys (r: 28–83)	USA
**SEGUE Framework**	Makoul (2001)	1: Rehabilitation Institute of Chicago training program	1: 16 video-consultations	USA
		2: Northwestern University Medical School	2: 52 video-consultations	
		3: different specialist physicians	3: 46 audio-records	
		4: primary care	4: 500 video-consultations (approx. 25 patients for each of the 20 academic general internists)	
**LIV-MAAS**	Robinson et al. (2002)	general practices	1: p: n = 213, 38% m, ph: n = 15 GPs, 67% m, age 35 to 62 ys, interviews	UK
	Enzer et al. (2003)	general practices	2: p: n = 71, 46% m, age 18 to>75 ys; ph: n = 8, 50% m, age 35 to 60 ys, video-consultation	UK
**VR-MICS**	Del Piccolo et al. (2004)	GPs practices	p: n = 238, 31.1% m, mean age 45 ys; ph: n = 6, 100% m, mean age 46 ys	Italy
	Del Piccolo et al. (2005)	GPs practices	UK p: n = 30, 40% m, mean age 45.7 ys; Italy p: n = 30, 40% m, mean age 44.1 ys; ph: n = 6 (UK)/6 (Italy), 100% m, age (r: 35–55) ys;	UK, Italy
**4HCS**	Krupat et al. (2006)	ambulatory care center of a teaching hospital	p: n = 100, 50% m, mean age 60 ys; p: n = 50; video-consultations	USA
	Fossli et al. (2010)	outpatient clinic and emergency department of a teaching hospital	p: n = 497, 48% male, mean age 46 ys (SD = 25); ph: n = 71, 62% m, mean age 41 ys (SD = 9); video-consultation	Norway
	Scholl et al. (2014)	outpatient care sample of a cross-sectional study	p: n = 67, 37.3% m, mean age 55.7 ys (SD = 15.34); ph: n = 22, 54.5% m, mean age 48.7 ys (SD = 8.22); audio-records	Germany
**PBCI**	Zandbelt et al. (2005)	outpatient division of an academic teaching hospital	p: n = 330, 42% m, mean age 53 ys (SD = 16), ph: n = 30, 53% male, 38 ys (SD = 8); video-consultations	Netherlands
**CAT**	Makoul et al. (2007)	clinical practices	1: p: n = 30; ph: n = 17; 2: p: n = 600; ph: n = 20; 3: p: n = 950, age from children to ≥75 ys; ph: n = 38	USA
**MPI**	Campbell et al. (2007)	GPs practices	p: n = 1884, 33.2% m, 7% not specified; ph: n = 91	Canada
**TCom-skill GP Scale**	Baumann et al. (2008)	medical check-up in preventive medical centre	p: n = 393, 50.4% m, mean age 47 ys (SD = 14)	France
**4HPQ**	Gulbrandsen et al. (2008)	hospitals	p: n = 210, 27% m, age (r: 23≥69) ys; ph: n = 16, 69% m, age (r: 29–61) ys	Norway
	Fossli et al (2011)	outpatient clinic and emergency department of a teaching hospital	p: n = 497, 48% m, mean age 46 ys (SD = 25); ph: n = 71, 62% m, mean age 41 ys (SD = 9)	Norway
**CASC**	Katsuyama et al. (2008)	physicians practice	p: n = 29, 17% m, mean age 37.9 ys (SD = 20.4); ph: n = 1; 29 audio-records	Japan
**QQPPI**	Bieber et al. (2010)	4 outpatient clinics of the Medical University Hospital of Heidelberg	p: n = 147, 44.9% m, mean age 48.8 ys (SD = 14,7), ph: n = 19, 68% m	Germany
**SCCAP**	Siminoff et al. (2011)	1: breast cancer patients and oncologists	p: n = 420, audio-records	USA
		2: audiotapes from requests for tissue from the families of deceased	patients 50 from 1200 audio-records	
		3: breast cancer patient and oncologists	p: n = 180, ph: n = 39, patient family members: n = 137	
**GPFI**	McMillan et al. (2011)	GPs practice	p: n = 18, ph: n = 6, video-consultations	UK
**PHCPCS**	Salt et al. (2012)	rheumatology clinic	p: n = 150, 26% male, mean age 54 ys (SD = 13.9; range: 21–83)	USA
**PPCB**	Wachira et al. (2013)	routine medical visits	p: n = 400, 43,5% m, mean age 39,5 ys (SD = 8.95; r: 19–73)	Kenya
**GCRS**	Burt et al. (2014)	physicians with simulated patients	n = 42 video-consultations	UK

*some studies used different settings/samples. p = patients, ph = physician, m = male, f = female, ys = years, r = range. Full titles of the measurements: Physicians-patient communication patterns (PPCP), Classification System of Byrne and Long (CSBL), Roter Interaction Analysis System (RIAS), Medical Communication Competence Scale (MCCS), SEGUE framework, LIV-MAAS Scale (LIV-MAAS), Verona Medical Interview Classification System (VR-MICS), Four Habits Coding Scheme (4HCS), Patient-centred behaviour coding instrument (PBCI), Communication Assessment Tool (CAT), Matched-pair instrument (MPI), TCom-skill GP Scale, the Four Habit Patient Questionnaire (4HPQ), the Computer Analysis system of the physician patient consultation process (CASC), Questionnaire on quality of Physician-Patient Interaction (QQPPI), Siminoff Communication Content & Affect Program (SCCAP), Generic peer feedback instrument (GPFI), the Patient–health care provider communication scale (PHCPCS), Assessment of a Physician-Patient Communication Behaviors Scale (PPCBS), Global Consultation Rating Scale (GCRS).

### Characteristics of included instruments

In total, we included 20 measures in the review. Four measures were not clearly named by the authors; we therefore used the description from the title or abstract to abbreviate the instruments in our description, the *Physicians-patient communication patterns (PPCP)* [46], *the Classification System of Byrne and Long (CSBL)* [23], *the Matched-pair instrument (MPI)* [48] and *the Generic peer feedback instrument (GPFI)* [46]. We found eleven measures that use observer coding or rating systems [23]–[25], [29], [30], [34], [40], [42], [43], [46], [47]. Five measures are patient-reported [32], [33], [39], [45], [50]. Another two instruments use both physician- and patient-reports [44], [48]. Only one measure solely measures the physician's rating [28] and a last measure is a computer based analysis [49]. Characteristics of the identified measures are displayed in Table 3.

**Table 3:** Characteristics of physician–patient communication measures.

Measure	Initial study/Authors (year)	Method/Viewpoint	Language	Dimensions/Scales	Items/Categories	Response
**PPCP**	Shapiro et al. (1981)	Observer rating system	English	5 scales	16 items	6-point scale
**CSBL**	Buijs et al. (1984)*	Observer coding system	Dutch	n/r	45/50 categories	4-point scale
**RIAS**	Ong et al. (1998)*	Observer coding/rating system	English, Dutch	4 content areas of behavioural categories, 6 global affect scales	34 categories (ph), 28 categories (p)	categories coded when they occur, 6-point scale
**MCCS**	Cegala et al. (1998)	Physician-reported	English	4 dimensions	25 items	7-point Likert Scale
**SEGUE Framework**	Makoul (2001)	Observer coding system	English	5 content areas	25 items	nominal scale
**LIV-MAAS**	Robinson et al. (2002)	Observer rating system	English	6 or 7 subscales	95 items	nominal scale, 3-point scale
**VR-MICS**	Del Piccolo et al. (2004)*	Observer coding system	Italian, English	7 dimensions	22 items (physician), 21 items (patient)	n/r
**4HCS**	Krupat et al. (2006)	Observer coding system	English, German	n/r	23 items	5-point Likert scale
**PBCI**	Zandbelt et al. (2005)	Observer coding system	Dutch	bi-dimensional, 3 content areas	19 items	behavior and content area coded when they occur
**CAT**	Makoul et al. (2007)	Patient- and physician-reported	English	uni-dimensional	15 items	5-point Likert scale
**MPI**	Campbell et al. (2007)	Patient- and physician-reported	English	bi-dimensional	19 items	5-point Likert scale
**TCom-skill GP Scale**	Baumann et al. (2008)	Patient-reported	English French	uni-dimensional	15 items	9-point scale
**4HPQ**	Gulbrandsen et al. (2008)	Patient-reported	Norwegian	uni-dimensional	10 items	4-point Likert scale
**CASC**	Katsuyama et al. (2008)	Computer analysis system	Japanese	n/r	n/r	n/r
**QQPPI**	Bieber et al. (2010)	Patient-reported	German	uni-dimensional	14 items	5-point Likert scale
**SCCAP**	Siminoff et al. (2011)	Observer coding/rating computer-based system	English	12 content themes, 23 communication behaviours, 8 observer ratings	n/r	most frequency, 7-point scales
**GPFI**	McMillan et al. (2011)	Observer rating system	English	n/r	24 items	7-point Likert scale
**PHCPCS**	Salt et al. (2012)	Patient-reported	English	bi-dimensional	21 items	4-point Likert scale
**PPCBS**	Wachira et al. (2013)	Patient-reported	Swahili, English	uni-dimensional, 2 subscales	13 items	5-point Likert scale
**GCRS**	Burt et al. (2014)	Observer rating system	English	n/r	12 items	3-point scale

*further study, no initial study included. p = patient, ph = physician, n/r = not reported. Full titles of the measurements: Physicians-patient communication patterns (PPCP), Classification System of Byrne and Long (CSBL), Roter Interaction Analysis System (RIAS), Medical Communication Competence Scale (MCCS), SEGUE framework, LIV-MAAS Scale (LIV-MAAS), Verona Medical Interview Classification System (VR-MICS), Four Habits Coding Scheme (4HCS), Patient-centred behaviour coding instrument (PBCI), Communication Assessment Tool (CAT), Matched-pair instrument (MPI), TCom-skill GP Scale, the Four Habit Patient Questionnaire (4HPQ), the Computer Analysis system of the physician patient consultation process (CASC), Questionnaire on quality of Physician-Patient Interaction (QQPPI), Siminoff Communication Content & Affect Program (SCCAP), Generic peer feedback instrument (GPFI), the Patient–health care provider communication scale (PHCPCS), Assessment of a Physician-Patient Communication Behaviors Scale (PPCBS), Global Consultation Rating Scale (GCRS).

### Methodological quality of the included studies

The results of COSMIN ratings are displayed in Table 4. Not all studies reported on all psychometric properties; thus, not each COSMIN criterion could be applied for each study. The studies assessed a median of 3 out of the nine COSMIN criteria. None of the included studies used the Item-Response-Theory. Internal consistency (Box A) was reported for fourteen studies [28], [32], [33], [37], [39], [41]–[48], [50]. Only two studies received an excellent [42] or good score [33] respectively, while the other studies received either a fair [32], [39], [44], [45], [50] or a poor score [28], [41], [43], [44], [46]–[48].

**Table 4:** Assessment of the methodological quality with COSMIN criteria.

Measure	Authors (Year)	Psychometric properties								
		A	B	C	D	E	F	G	H	I
**PPCP**	Shapiro et al., (1981)	0	+ ^b^		0					
**CSBL**	Buijs et al. (1984)		+ ^b^							
**RIAS**	Ong et al., 1998		0 ^b^		0		0			
**MCCS**	Cegala et al. (1998)	0			+	0	0			
**SEGUE Framework**	Makoul (2001)		++^ b^, 0^ c^		+++					
**LIV-MAAS**	Robinson et al. (2002)				0					
	Enzer et al. (2003)		0^ b^,++ ^b^							
**VR-MICS**	Del Piccolo et al. (2004)					+++	+			
	Del Piccolo et al. (2005)		+^ b, c^					0		
**4HCS**	Krupat et al. (2006)	0	0 ^b^		0		0			
	Fossli et al. (2010)		0 ^b^							
	Scholl et al. (2014)	0	++ ^b^,+ ^c^			0		0^d^		
**PBCI**	Zandbelt et al. (2005)	+++	++ ^b^		0	+++	++^p^/0^ph^			
**CAT**	Makoul et al. (2007)	+			++	+				
**MPI**	Campbell et al. (2007)	0			0	+	+			
**TCom-skill GP Scale**	Baumann et al. (2008)	+	+ ^a^		0	+				
**4HPQ**	Gulbrandsen et al. (2008)	+	+ ^b^		0	+	0	0^d^		
	Fossli et al (2011)	0					+			
**CASC**	Katsuyama et al. (2008)				0		0			
**QQPPI**	Bieber et al. (2010)	++	0 ^a^		0	++	0			
**SCCAP**	Siminoff et al. (2011)	0	0 ^b^		0		0			
**GPFI**	McMillan et al. (2011)		0 ^b^		0					
**PHCPCS**	Salt et al. (2012)	+	+ ^a^		0	+	0			
**PPCB**	Wachira et al. (2013)	+	0 ^a^		0	+		+^d^		
**GCRS**	Burt et al. (2014)		+		0					

COSMIN psychometric property boxes: A =  internal consistency, B =  reliability, C =  measurement error, D =  content validity, E =  structural validity, F =  hypotheses testing, G =  cross-cultural validity, H =  criterion validity, I =  responsiveness. 4-point scale rating: +++ =  excellent, ++ =  good, + =  fair, 0 =  poor, empty space  =  COSMIN rating not applicable. For exact information regarding the definitions of psychometric properties and 4-point scale rating see COSMIN website (www.cosmin.nl). ^a^  =  Test-Retest-Reliability, ^b^  =  Inter-rater-Reliability, ^c^  =  Intra-rater-Reliability, ^d^  =  only evaluation of the quality of the translation procedure,^ p^  =  patient version, ^ph^  =  physician version.

The second COSMIN box, Reliability (Box B), could be applied to eighteen studies [23], [25], [29], [30], [32]–[36], [38], [39], [41]–[43], [45]–[47], [50]. This box was particularly relevant for the observer instruments, which in many cases reported on inter-rater- or/and intra-rater-reliability. Fourteen studies reported on one form of reliability and received one score for box B. One study received a good score [42], six studies received a fair score [23], [32], [34], [39], [45], [46] and seven studies received a poor score [25], [30], [33], [38], [43], [47], [50]. Three studies reported on two forms of reliability and therefore received two scores for box B. Makoul [29] received a good score for inter-rater-reliability and a poor score for intra-rater-reliability. Del Piccolo et al. [35] scored fair for both inter-rater-reliability and intra-rater-reliability. Scholl et al. [41] were rated good for inter-rater-reliability and fair for intra-rater-reliability. Enzer et al. [36] used two samples to examine reliability. This study received two scores, poor for the first sample and good for the second sample.

Measurement error (Box C) was not reported in any of the studies. The content validity box (Box D) was applied to all studies that were conducted on the initial development of the measures. Thus, eighteen studies were rated [25], [28]–[30], [32]–[34], [39], [40], [42]–[50]. The majority of the studies scored poorly [25], [30], [32]–[34], [39], [40], [42], [43], [45]–[50]. The study on the SEGUE framework was the only one that was rated as excellent [29], while two studies were rated as either good [44] or fair [28]. Eleven studies assessed structural validity (Box E) [24], [28], [32], [33], [39], [41], [42], [44], [45], [48], [50]. Two studies [24], [42] were rated as excellent, one study scored good [33], six studies [32], [39], [44], [45], [48], [50] scored fair and two studies [28], [41] received a poor score. Hypotheses testing rating (Box F) was assessed in twelve studies [24], [25], [28], [33], [37], [39], [42], [43], [45], [47]–[49]. Three studies were rated as fair [24], [37], [48], eight studies received a poor score only [25], [28], [33], [39], [43], [45], [47], [49]. The study on the PCBI [42] received a good rating for the physician scale and a poor rating for its patient scale.

Cross-cultural validity (Box G) was only assessed in one study [35] and rated as poor. Three studies [39], [41], [50] translated instruments, but did not assess cultural validity. For these studies, the translation procedure was rated with the items 4 to 11 of Box G. Criterion validity (Box H) and Responsiveness (Box I) were not analyzed by any of the studies. The detailed COSMIN ratings on item level are shown in S1 Table and S2 Table in S2 File.

### Quality of psychometric properties

The evaluation of the quality of psychometric properties of the identified measures was conducted with the criteria of Terwee et al. and results are shown in Table 5. Content validity received a positive score in eight studies [28], [29], [32], [33], [40], [42], [44], [45]. Four studies [25], [46], [48], [50] were rated as intermediate, and six studies [30], [34], [39], [43], [47], [49] received a negative rating. The other studies did not give any information on content validity. For internal consistency, positive ratings were found for seven studies [32], [33], [37], [39], [44], [45], [50], six studies received intermediate ratings [28], [41], [43], [46]–[48] and one study received a negative score [42]. For half of the studies, no information was available on internal consistency. The majority of the studies also did not provide information on construct validity. Nevertheless, five studies received a positive score [28], [33], [37], [42], [45] and five studies an intermediate score [24], [39], [43], [47], [49]. Two studies scored negative on construct validity [25], [48]. Information on reproducibility (reliability) was rated as positive for five studies [29], [32], [35], [38], [41], intermediate for ten studies [23], [25], [30], [33], [34], [39], [43], [46], [47], [50], and negative for one study [45]. The study on the LIV-MAAS [36] was rated as positive and intermediate, because this study examined reliability in two different samples and therefore one rating for each sample was conducted. Scores were positive and negative for the study on the PBCI [42] due to its two dimensions (1.facilitating, 2. inhibiting). Scorings on interpretability were either intermediate or no information was available. None of the studies gave information on criterion validity, reproducibility (agreement), responsiveness or floor and ceiling effects.

**Table 5:** Quality rating of psychometric properties with Terwee et al.'s criteria.

Measure	Authors (Year)	Psychometric properties								
		Content validity	Internal consistency	Criterion validity	Construct validity	Reproducibility (Agreement)	Reproducibility (Reliability)	Responsiveness	Floor & ceiling effects	Interpretability
**PPCP**	Shapiro et al., (1981)	?	?	0	0	0	?	0	0	?
**CSBL**	Buijs et al. (1984)	0	0	0	0	0	?	0	0	0
**RIAS**	Ong et al., 1998	?	0	0	-	0	?	0	0	?
**MCCS**	Cegala et al. (1998)	+	?	0	+	0	0	0	0	?
**SEGUE Framework**	Makoul (2001)	+	0	0	0	0	+	0	0	?
**LIV-MAAS**	Robinson et al. (2002)	+	0	0	0	0	0	0	0	0
	Enzer et al. (2003)	0	0	0	0	0	?^1^/+^2^	0	0	?
**VR-MICS**	Del Piccolo et al. (2004)	0	0	0	?	0	0	0	0	?
	Del Piccolo et al. (2005)	0	0	0	0	0	+	0	0	?
**4HCS**	Krupat et al. (2006)	-	?	0	?	0	?	0	0	0
	Fossli et al. (2010)	0	0	0	0	0	+	0	0	?
	Scholl et al. (2014)	0	?	0	0	0	+	0	0	?
**PBCI**	Zandbelt et al. (2005)	+	-	0	+	0	+ ^f^/− ^i^	0	0	?
**CAT**	Makoul et al. (2007)	+	+	0	0	0	0	0	0	?
**MPI**	Campbell et al. (2007)	?	?	0	-	0	0	0	0	0
**TCom-skill GP Scale**	Baumann et al. (2008)	+	+	0	0	0	+	0	0	?
**4HPQ**	Gulbrandsen et al. (2008)	-	+	0	?	0	?	0	0	?
	Fossli et al (2011)	0	+	0	+	0	0	0	0	0
**CASC**	Katsuyama et al. (2008)	-	0	0	?	0	0	0	0	?
**QQPPI**	Bieber et al. (2010)	+	+	0	+	0	?	0	0	?
**SCCAP**	Siminoff et al. (2011)	-	?	0	?	0	?	0	0	0
**GPFI**	McMillan et al. (2011)	-	0	0	0	0	?	0	0	?
**PHCPCS**	Salt et al. (2012)	+	+	0	+	0	-	0	0	0
**PPCB**	Wachira et al. (2013)	?	+	0	0	0	?	0	0	?
**GCRS**	Burt et al. (2014)	-	0	0	0	0	?	0	0	0

Rating: + =  positive, ? =  intermediate, − =  negative, 0 =  no information available. For exact information regarding the definitions of psychometric properties see [21]. ^1^  =  first sample, ^2^  =  second sample. f =  first dimension (facilitating), i =  second dimension (inhibiting).

## Discussion

This review sought to systematically examine studies on psychometric properties of measures on physician-patient communication, to investigate the methodological quality of these studies and to evaluate the quality of the psychometric properties of the identified measures. We extracted data from 25 studies examining 20 measures of physician-patient communication.

Regarding the methodical quality of the studies, the results revealed a heterogeneous picture. Only two studies received an excellent or good score on internal consistency [33], [42]. For reliability, the best rating was good for four of the studies [29], [36], [41], [42]. Six studies [29], [36], [38], [40], [41], [43] showed conflicting results. For content validity, two studies received an excellent or good score [29], [44]. From the studies that investigated structural validity, three were rated of good or excellent quality [24], [33], [42]. For hypothesis testing, only one study [42] received a good score. Cross-cultural validity was only examined for one measure [35] which scored poor. In summary, three of the instruments received poor scores on the overall COSMIN rating. The study on the PBCI [42] tested the most psychometric properties and was the only study that achieved two excellent and two good scores.

Remarkably, none of the studies on patient- or physician-reported measures received an excellent score on any psychometric property, but three of the observer ratings did. However, when ratings are examined for each study per item (see S1 Table and S2 Table in S2 File), most of the studies received more excellent and good scores. Furthermore, the items concerning the handling of missing items were rated on the COSMIN checklist for the patient- or physician-reported measures. In case of a low rating on these items and due to the COSMIN recommendation namely to count the worst score per box, the final results of the patient- or physician-reported measures might be lower than for the observer rating systems.

Quality of psychometric properties evaluated with the Terwee et al.'s criteria [21] were available for content validity, internal consistency, construct validity, reproducibility (reliability) and interpretability. For criterion validity, reproducibility (agreement), responsiveness, floor- and ceiling effects none of the studies reported information. For measures that describe the absence or presence of certain communication aspects, reporting of floor and ceiling effects might be not appropriate on the item level. In the case of available information, psychometric properties scored mostly positive or intermediate. Negative ratings for the quality of content validity were found only for three studies [30], [47], [49]. Although some of the measures scored well on the methodical rating with COSMIN, the evaluation with the Terwee et al.'s criteria [21] showed clearly that the quality of the results was not always sufficient. For example, the study on the PBCI [42] scored excellent for the methodological assessment of internal consistency, but the quality of this psychometric property was only rated poor with the Terwee et al.'s criteria. The findings were similar for the study on the MPI [48] on construct validity.

In summary, the results for the methodological quality assessment show that studies reported on a median of 3 out of the nine COSMIN criteria. However, several flaws were revealed concerning the methodical quality and the quality of the psychometric properties. Content validity and hypothesis testing was of rather poor methodical quality and measurement error, criterion validity and responsiveness were almost not considered and should be addressed in future psychometric studies. The quality rating with Terwee et al.'s criteria showed that some measures received positive scores even though the methodological procedure was not always adequate. When combining the ratings of the studies on the COSMIN and Terwee et al.'s criteria, best results were received for the studies on the following measures: the SEGUE framework [29], the PBCI [42], and the QQPPI [33]. Each achieved at least two excellent or good ratings on COSMIN and two positive ratings on Terwee et al.'s criteria. The studies on the TCom-skill GP [32] scale and the PHCPCS [45] scored good and fair on COSMIN, but received only three positive ratings on the Terwee et al.'s rating.

Our results are barely comparable to the previous reviews conducted by Ong et al. [12] and Boon and Stewart [13]. Ong et al. [12] mainly presented an overview of measures of physician-patient communication without evaluating the psychometric properties of the instruments. Boon and Stewart [13] included instruments developed for the use in medical education settings, as well as manuals of measures without a validation-study published in a peer reviewed journal, therefore almost none of the measures included in that review were included in this current review. Moreover, since then several new instruments examining physician-patient communication were developed which could not be considered in those reviews, but were evaluated in this study.

From our results, we suggest to further evaluate psychometric properties of existing measures on physician-patient communication using more rigorous methodological designs. Furthermore, there is a particular need to conduct further psychometric evaluation studies on the measures, especially to assess psychometric properties that have not been tested yet (e.g. responsiveness). However, the results from this study can be helpful for researchers to select the most appropriate measure for conducting a study on physician-patient communication. Since the included measures have different rating perspectives, the selection of a measure over another will be driven by the study aim and the feasibility in a certain study setting.

### Strengths and limitations of the study

A strength of this review is the detailed electronic search strategy, which was based on the COSMIN filter [14]. Moreover, two researchers independently assessed all records and full texts and together with a third reviewer quality was ensured by double or triple assessment of some studies. Another notable strength is that quality assessments were conducted by using both the COSMIN checklist and the quality criteria for good psychometric properties developed by Terwee et al. [21]. To our knowledge, no systematic review on physician-patient communication to date provides an elaborated judgment on the methodical quality of the studies and their final results on the psychometric properties following the recommendation of Mokkink and colleagues [17].

However, the current review has several limitations that need to be addressed. First, our review includes only generic measures for reasons of feasibility; and measures developed specifically for the medical education context or specific indications were beyond the scope of this review. Second, our search was limited to studies published in German or English. Therefore, studies published in other languages may not have been included. Third, although we believe our search strategy was very sensitive and guided by methodological recommendations for systematic reviews [14], [51], not all studies were identified by our electronic search and were subsequently added from the authors' personal knowledge. However, the importance of personal knowledge as a valid source has been described in the literature [51]. Fourth, due to the lag between the time of the search completion and the final manuscript publication, we may have missed out recently published studies on this topic.

## Conclusion

This systematic review provides an overview on measures on physician-patient communication and helps researchers to identify the appropriate instrument for their research purpose. Moreover, our study highlighted current gaps in the methodological quality of studies on psychometric properties and the quality of their results. We recommend that future evaluation studies on psychometric properties should apply standards like the COSMIN checklist in order to enhance quality of the studies and to increase the comparison of results.

## Supporting Information

S1 File **Electronic database search strategy for EMBASE, PsycINFO Pubmed.**
(DOCX)

S2 File **S1 Table and S2 Table.** Detailed results for the COSMIN checklist with 4-point scale rating.
(DOCX)

S1 Checklist **PRISMA checklist.**
(DOC)
