# Supplementary material for: Measurement of Physician-Patient Communication—A Systematic Review
Source: PLoS One. 2014 Dec 22;9(12):e112637. doi: 10.1371/journal.pone.0112637 (PMC4273948; doi:10.1371/journal.pone.0112637)
Supplement: S1 File — Electronic database search strategy for EMBASE, PsycINFO Pubmed. (DOCX) [file pone.0112637.s001.docx]

**File S1: Electronic database search strategy for EMBASE, PsycInfo, Pubmed**

**EMBASE via Ovid**

Clinician (OR Physician/ Doctor/ practitioner/ health care provider/ provider) AND

Patient (OR Client) AND

Communication (OR verbal behavio?r/ non-verbal behavio?r/ interaction/ Interpersonal behavio?r/ Body language) AND

Measur* (OR Health Survey/ instrument/ test/ Scale*/ Questionnair*/ Assess*/ Rating/ Coding) AND

Exp Reliability (OR Exp Validity/ Valid*/ Accura*/ Reliab*/ Factor structure/ factor* analy*is/ Internal consistency/ Cronbach*/ Rasch analysis/ Item response/ Differential item function/ Responsiveness)

Limitations:

- English OR German

**PsycInfo via Ovid**

Clinician (OR Physician/ Doctor/ practitioner/ health care provider/ provider) AND

Patient (OR Client) AND

Communication (OR verbal behavio?r/ non-verbal behavio?r/ interaction/ Interpersonal behavio?r/ Body language) AND

Exp Measurement (OR Health Survey/ instrument/ test/ Measur*/ Index/ Indices/ Scale*/ Questionnair*/ Self Report*/ rating/ Assess*/ Coding) AND

Exp Test construction (OR Exp factor analysis/ Exp factor structure/ Exp test validity/ Exp statistical validity/ Exp statistical reliability/ Exp test reliability/ Exp interrater reliability/ Valid*/ Accura*/ Reliab*/ Consisten*/ factor*/ Factor structure/ factor* analy*is/ Internal consistency/ Test-retest/ Cronbach*/ Rasch analysis/ Item response/ Differential item function/ Item reduction/ Kappa/ Responsiveness/ Intraclass correlates)

Limitations:

- English OR German
- adulthood <18+ years> (OR not categorised)
- "0100 journal" OR "0110 peer-reviewed journal" OR “0120 non-peer-reviewed journal" OR "0130 peer-reviewed status unknown"(OR not categorised)

**PubMed via Ovid**

Clinician (OR Physician/ Doctor/ practitioner/ health care provider/ provider) AND

Patient (OR Client) AND

Communication (“verbal behaviour”/ “verbal behavior”/ “non-verbal behaviour”/ “non-verbal behavior”/ interaction/ “Interpersonal behavior”/ “Interpersonal behaviour”) AND

COSMIN filter and COSMIN Exclusion Filter (see reference)

Limitations:

- English OR German
- "all adult"[Filter (OR not categorised)

**COSMIN-Filter**

Terwee, C. B., Jansma, E. P., Riphagen, I. I., & de Vet, H. C. (2009). Development of a methodological PubMed search filter for finding studies on measurement properties of measurement instruments. Quality of Life Research, 18(8), 1115-1123.

Source: http://link.springer.com/article/10.1007%2Fs11136-009-9528-5/fulltext.html
