# Supplementary material for: Measurement of Physician-Patient Communication—A Systematic Review
Source: PLoS One. 2014 Dec 22;9(12):e112637. doi: 10.1371/journal.pone.0112637 (PMC4273948; doi:10.1371/journal.pone.0112637)
Supplement: S2 File — S1 Table and S2 Table. Detailed results for the COSMIN checklist with 4-point scale rating. (DOCX) [file pone.0112637.s002.docx]

**Table S1: Detailed results for the COSMIN checklist with 4-point scale rating (Studies C1 to 12)**

| *Box* | *COSMIN psychometric properties/Items** |  |  |  |  |  |  |  |  |  |  |  |  |
| --- | --- | --- | --- | --- | --- | --- | --- | --- | --- | --- | --- | --- | --- |
|  |  | **C1** | **C2** | **C3** | **C4** | **C5** | **C6** | **C7** | **C8** | **C9** | **C10** | **C11** | **C12** |
| **IRT** | **IRT** |  |  |  |  |  |  |  |  |  |  |  |  |
| **A** | **Internal consistency** | **C1** |  |  | **C4** |  |  |  |  |  | **C10** |  | **C12** |
| 1 | Does scale consist of effect indicators, i.e. is it based on a reflective model? | ok |  |  | ok |  |  |  |  |  | ok |  | ok |
| 2 | Percentage of missing items given? | n/a |  |  | ++ |  |  |  |  |  | n/a |  | n/a |
| 3 | Description of how missing items were handled? | n/a |  |  | + |  |  |  |  |  | n/a |  | n/a |
| 4 | Sample size included in internal consistency analysis adequate? | 0 |  |  | ++ |  |  |  |  |  | +++ |  | ++ |
| 5 | Unidimensionality of scale checked, i.e. factor analysis or IRT model applied? | 0 |  |  | 0 |  |  |  |  |  | 0 |  | 0 |
| 6 | Sample size included in unidimensionality analysis adequate? | n/a |  |  | n/a |  |  |  |  |  | n/a |  | n/a |
| 7 | Internal consistency statistic calculated for each scale separately? | +++ |  |  | +++ |  |  |  |  |  | +++ |  | +++ |
| 8 | Any important flaws in design or method of the study? | +++ |  |  | +++ |  |  |  |  |  | +++ |  | +++ |
| 9 | For CTT, continuous scores: Cronbach's alpha calculated? | +++ |  |  | +++ |  |  |  |  |  | +++ |  | +++ |
| 10 | For CTT, dichotomous scores: Cronbach's alpha or KR-20 calculated? | n/a |  |  | n/a |  |  |  |  |  | n/a |  | n/a |
| 11 | For IRT, goodness of fit statistic at global level calculated? | n/a |  |  | n/a |  |  |  |  |  | n/a |  | n/a |
|  | **Final score Box A** | **0** |  |  | **0** |  |  |  |  |  | **0** |  | **0** |
| **B** | **Reliability** | **C1^b^** | **C2^b^** | **C3^b^** |  | **C5^b,c^** |  | **C7^b^** |  | **C9^b,c^** | **C10^b^** | **C11^b^** | **C12^b,c^** |
| 1 | Percentage of missing items given? | n/a | n/a | n/a |  | n/a |  | n/a |  | n/a | n/a | n/a | n/a |
| 2 | Description of how missing items were handled? | n/a | n/a | n/a |  | n/a |  | n/a |  | n/a | n/a | n/a | n/a |
| 3 | Sample size included in analysis adequate? | ++ | + | 0 |  | ++**^b^**/0**^c^** |  | 0^1^/++^2^ |  | + | 0 | 0 | ++**^b^**/+**^c^** |
| 4 | At least two measurements available? | +++ | +++ | +++ |  | +++ |  | +++ |  | +++ | +++ | +++ | +++ |
| 5 | Administrations independent? | ++ | ++ | +++ |  | ++ |  | +++ |  | +++ | +++ | +++ | ++ |
| 6 | Time interval stated? | n/a | n/a | n/a |  | n/a**^b^**/+**^c^** |  | n/a |  | n/a**^b^**/+++**^c^** | n/a | n/a | n/a**^b^**/+++**^c^** |
| 7 | Patients stable in the interim period on the construct to be measured? | n/a | n/a | n/a |  | n/a |  | n/a |  | n/a | n/a | n/a | n/a |
| 8 | Time interval appropriate? | n/a | n/a | n/a |  | n/a |  | n/a |  | n/a**^b^**/+++**^c^** | n/a | n/a | n/a**^b^**/+++**^c^** |
| 9 | Test conditions similar for both measurements? | ++ | ++ | ++ |  | ++ |  | ++ |  | ++ | ++ | ++ | ++ |
| 10 | Any important flaws in design or methods of the study? | +++ | +++ | +++ |  | +++ |  | +++ |  | +++ | +++ | +++ | +++ |
| 11 | For continuous scores: intraclass correlation coefficient (ICC) calculated? | + | + | + |  | n/a |  | ++ |  | ++ | + | + | +++ |
| 12 | For dichotomous/nominal/ordinal scores: kappa calculated? | n/a | n/a | n/a |  | +++ |  | n/a |  | n/a | n/a | n/a | n/a |
| 13 | For ordinal scores: weighted kappa calculated? | n/a | n/a | n/a |  | n/a |  | n/a |  | n/a | n/a | n/a | n/a |
| 14 | For ordinal scores: weighting scheme described? | n/a | n/a | n/a |  | n/a |  | n/a |  | n/a | n/a | n/a | n/a |
|  | **Final score Box B** | **+** | **+** | **+** |  | **++/0** |  | **0/++** |  | **+** | **0** | **0** | **++/+** |
| **C** | **Measurement error** |  |  |  |  |  |  |  |  |  |  |  |  |

… Continued on the next page

| **D** | **Content validity** | **C1** |  | **C3** | **C4** | **C5** | **C6** |  |  |  | **C10** |  |  |
| --- | --- | --- | --- | --- | --- | --- | --- | --- | --- | --- | --- | --- | --- |
| 1 | Assessment of whether all items refer to relevant aspects of the construct? | 0 |  | +++ | +++ | +++ | +++ |  |  |  | 0 |  |  |
| 2 | Assessment of whether all items are relevant for the study population | 0 |  | 0 | +++ | +++ | +++ |  |  |  | 0 |  |  |
| 3 | Assessment of whether all items are relevant for the purpose of instrument | + |  | + | + | +++ | + |  |  |  | +++ |  |  |
| 4 | Assessment of whether all items together reflect the construct? | 0 |  | +++ | + | +++ | 0 |  |  |  | +++ |  |  |
| 5 | Any important flaws in design or method of the study? | +++ |  | +++ | +++ | +++ | +++ |  |  |  | +++ |  |  |
|  | **Final score Box D** | **0** |  | **0** | **+** | **+++** | **0** |  |  |  | **0** |  |  |
| **E** | **Structural validity** |  |  |  | **C4** |  |  |  | **C8** |  |  |  | **C12** |
| 1 | Does scale consist of effect indicators, i.e. is it based on a reflective model? |  |  |  | ok |  |  |  | ok |  |  |  | ok |
| 2 | Percentage of missing items given? |  |  |  | ++ |  |  |  | n/a |  |  |  | n/a |
| 3 | Description of how missing items were handled? |  |  |  | + |  |  |  | n/a |  |  |  | n/a |
| 4 | Sample size included in analysis adequate? |  |  |  | 0 |  |  |  | +++ |  |  |  | 0 |
| 5 | Any important flaws in design or method of the study? |  |  |  | +++ |  |  |  | +++ |  |  |  | 0 |
| 6 | For CTT: exploratory or confirmatory factor analysis performed? |  |  |  | 0 |  |  |  | +++ |  |  |  | 0 |
| 7 | For IRT: tests for determining (uni-) dimensionality of the items performed? |  |  |  | n/a |  |  |  | n/a |  |  |  | n/a |
|  | **Final score Box E** |  |  |  | **0** |  |  |  | **+++** |  |  |  | **0** |
| **F** | **Hypotheses testing** |  |  | **C3** | **C4** |  |  |  | **C8** |  | **C10** |  |  |
| 1 | Percentage of missing items given? |  |  | n/a | ++ |  |  |  | n/a |  | ++ |  |  |
| 2 | Description of how missing items were handled? |  |  | n/a | + |  |  |  | n/a |  | + |  |  |
| 3 | Sample size included in analysis adequate? |  |  | 0 | 0 |  |  |  | +++ |  | 0 |  |  |
| 4 | Hypotheses regarding correlations or mean differences formulated a priori? |  |  | +++ | +++ |  |  |  | + |  | 0 |  |  |
| 5 | Expected direction of correlations or mean differences included in hypotheses? |  |  | +++ | +++ |  |  |  | ++ |  | ++ |  |  |
| 6 | Expected absolute/relative magnitude of correlations/mean differences included in hypotheses? |  |  | ++ | ++ |  |  |  | ++ |  | ++ |  |  |
| 7 | For convergent validity: adequate description provided of comparator instrument(s)? |  |  | n/a | n/a |  |  |  | n/a |  | +++ |  |  |
| 8 | For convergent validity: measurement properties of comparator instrument(s) adequately described? |  |  | n/a | n/a |  |  |  | n/a |  | 0 |  |  |
| 9 | Any important flaws in design or method of the study? |  |  | +++ | +++ |  |  |  | +++ |  | +++ |  |  |
| 10 | Design and statistical methods adequate for hypotheses to be tested? |  |  | +++ | +++ |  |  |  | +++ |  | ++ |  |  |
|  | **Final score Box F** |  |  | **0** | **0** |  |  |  | **+** |  | **0** |  |  |

… Continued on the next page

| **G** | **Cross-cultural validity** |  |  |  |  |  |  |  |  | **C9** |  |  |  | **C12** |
| --- | --- | --- | --- | --- | --- | --- | --- | --- | --- | --- | --- | --- | --- | --- |
| 1 | Percentage of missing items given? |  |  |  |  |  |  |  |  | n/a |  |  |  | n/a |
| 2 | Description of how missing items were handled? |  |  |  |  |  |  |  |  | n/a |  |  |  | n/a |
| 3 | Sample size included in analysis adequate? |  |  |  |  |  |  |  |  | 0 |  |  |  | n/a |
| 4 | Both the original language in which instrument was developed and language in which instrument was translated described? |  |  |  |  |  |  |  |  | +++ |  |  |  | +++ |
| 5 | Expertise of people involved in translation process adequately described? |  |  |  |  |  |  |  |  | +++ |  |  |  | + |
| 6 | Did translators work independently from each other? |  |  |  |  |  |  |  |  | ++ |  |  |  | +++ |
| 7 | Items translated forward and backward? |  |  |  |  |  |  |  |  | 0 |  |  |  | 0 |
| 8 | Adequate description of how differences between the original and translated versions were resolved? |  |  |  |  |  |  |  |  | ++ |  |  |  | +++ |
| 9 | Translation reviewed by a committee? |  |  |  |  |  |  |  |  | +++ |  |  |  | +++ |
| 10 | Instrument pre-tested (e.g. cognitive interviews) to check interpretation, cultural relevance of the translation, and ease of comprehension? |  |  |  |  |  |  |  |  | 0 |  |  |  | 0 |
| 11 | Sample used in the pre-test adequately described? |  |  |  |  |  |  |  |  | 0 |  |  |  | n/a |
| 12 | Samples similar for all characteristics except language and/or cultural background? |  |  |  |  |  |  |  |  | +++ |  |  |  | n/a |
| 13 | Any important flaws in design or methods of the study? |  |  |  |  |  |  |  |  | +++ |  |  |  | n/a |
| 14 | For CTT: confirmatory factor analysis performed? |  |  |  |  |  |  |  |  | 0 |  |  |  | n/a |
| 15 | For IRT: differential item function (DIF) between language groups assessed? |  |  |  |  |  |  |  |  | n/a |  |  |  | n/a |
|  | **Final score Box G** |  |  |  |  |  |  |  |  | **0** |  |  |  | **0** |
| **H** | **Criterion validity** |  |  |  |  |  |  |  |  |  |  |  |  |  |
| **I** | **Responsiveness** |  |  |  |  |  |  |  |  |  |  |  |  |  |

* Description of item content altered to fit this table. For exact item content see COSMIN website (www.cosmin.nl) . Study IDs: C1 = Shapiro et al., 1981; C2 = Buijs et al., 1984; C3 = Ong et al., 1998; C4 = Cegala et al., 1998; C5 = Makoul, 2001; C6 = Robinson et al., 2002; C7 = Enzer et al., 2003; C8 = Del Piccolo et al., 2004; C9 = Del Piccolo et al., 2005; C10 = Krupat et al., 2006; C11 = Fossli et al., 2010; C12 = Scholl et al., 2014. 4-point scale rating: +++ = excellent, ++ = good, + = fair, 0 = poor, empty space = COSMIN rating not applicable. n/a = not applicable. Box B Reliability: a = Retest-Reliability, b = Interrater-Reliability, c = Intrarater-Reliability. If two reliability coefficients were calculated the rating refers to both, unless otherwise indicated. 1=first sample, 2=second sample.

**Table S2: Detailed results for the COSMIN checklist with 4-point scale rating (Studies C13 to C25)**

| *Box* | *COSMIN psychometric properties/Items** |  |  |  |  |  |  |  |  |  |  |  |  |  |
| --- | --- | --- | --- | --- | --- | --- | --- | --- | --- | --- | --- | --- | --- | --- |
|  |  | **C13** | **C14** | **C15** | **C16** | **C17** | **C18** | **C19** | **C20** | **C21** | **C22** | **C23** | **C24** | **C25** |
| **IRT** | **IRT** |  |  |  |  |  |  |  |  |  |  |  |  |  |
| **A** | **Internal consistency** | **C13** | **C14** | **C15** | **C16** | **C17** | **C18** |  | **C20** | **C21** |  | **C23** | **C24** |  |
| 1 | Does scale consist of effect indicators, i.e. is it based on a reflective model? | ok | ok | ok | ok | ok | ok |  | ok | ok |  | ok | ok |  |
| 2 | Percentage of missing items given? | n/a | ++ | +++ | +++ | +++ | +++ |  | ++ | n/a |  | + | ++ |  |
| 3 | Description of how missing items were handled? | n/a | + | + | + | + | +++ |  | +++ | n/a |  | +++ | + |  |
| 4 | Sample size included in internal consistency analysis adequate? | +++ | +++ | +++ | +++ | +++ | +++ |  | +++ | ++ |  | +++ | +++ |  |
| 5 | Unidimensionality of scale checked, i.e. factor analysis or IRT model applied? | +++ | +++ | +++ | +++ | +++ | 0 |  | +++ | 0 |  | +++ | +++ |  |
| 6 | Sample size included in unidimensionality analysis adequate? | +++ | +++ | +++ | +++ | +++ | n/a |  | +++ | 0 |  | + | +++ |  |
| 7 | Internal consistency statistic calculated for each scale separately? | +++ | +++ | 0 | +++ | +++ | 0 |  | +++ | 0 |  | +++ | +++ |  |
| 8 | Any important flaws in design or method of the study? | +++ | +++ | +++ | +++ | +++ | +++ |  | +++ | +++ |  | +++ | + |  |
| 9 | For CTT, continuous scores: Cronbach's alpha calculated? | +++ | +++ | +++ | +++ | +++ | +++ |  | +++ | +++ |  | +++ | +++ |  |
| 10 | For CTT, dichotomous scores: Cronbach's alpha or KR-20 calculated? | n/a | n/a | n/a | n/a | n/a | n/a |  | n/a | n/a |  | n/a | n/a |  |
| 11 | For IRT, goodness of fit statistic at global level calculated? | n/a | n/a | n/a | n/a | n/a | n/a |  | n/a | n/a |  | n/a | n/a |  |
|  | **Final score Box A** | **+++** | **+** | **0** | **+** | **+** | **0** |  | **++** | 0 |  | **+** | **+** |  |
| **B** | **Reliability** | **C13^b^** |  |  | **C16^a^** | **C17^b^** |  |  | **C20^a^** | **C21^b^** | **C22^b^** | **C23^a^** | **C24^a^** | **C25^b^** |
| 1 | Percentage of missing items given? | n/a |  |  | +++ | +++ |  |  | ++ | n/a | n/a | ++ | ++ | +++ |
| 2 | Description of how missing items were handled? | n/a |  |  | + | + |  |  | +++ | n/a | n/a | +++ | + | +++ |
| 3 | Sample size included in analysis adequate? | +++ |  |  | ++ | +++ |  |  | +++ | +++ | 0 | ++ | +++ | + |
| 4 | At least two measurements available? | +++ |  |  | +++ | +++ |  |  | +++ | +++ | +++ | +++ | +++ | +++ |
| 5 | Administrations independent? | ++ |  |  | ++ | +++ |  |  | ++ | +++ | ++ | ++ | ++ | +++ |
| 6 | Time interval stated? | n/a |  |  | +++ | n/a |  |  | +++ | n/a | n/a | +++ | +++ | n/a |
| 7 | Patients stable in the interim period on the construct to be measured? | n/a |  |  | + | n/a |  |  | ++ | n/a | n/a | ++ | ++ | n/a |
| 8 | Time interval appropriate? | n/a |  |  | +++ | n/a |  |  | +++ | n/a | n/a | +++ | 0 | n/a |
| 9 | Test conditions similar for both measurements? | ++ |  |  | + | +++ |  |  | 0 | ++ | ++ | ++ | +++ | +++ |
| 10 | Any important flaws in design or methods of the study? | +++ |  |  | +++ | +++ |  |  | +++ | +++ | +++ | + | +++ | +++ |
| 11 | For continuous scores: intraclass correlation coefficient (ICC) calculated? | +++ |  |  | +++ | ++ |  |  | + | 0 | 0 | ++ | + | +++ |
| 12 | For dichotomous/nominal/ordinal scores: kappa calculated? | n/a |  |  | n/a | n/a |  |  | n/a | n/a | n/a | n/a | n/a | n/a |
| 13 | For ordinal scores: weighted kappa calculated? | n/a |  |  | n/a | n/a |  |  | n/a | n/a | n/a | n/a | n/a | n/a |
| 14 | For ordinal scores: weighting scheme described? | n/a |  |  | n/a | n/a |  |  | n/a | n/a | n/a | n/a | n/a | n/a |
|  | **Final score Box B** | **++** |  |  | **+** | **+** |  |  | **0** | **0** | **0** | **+** | **0** | **+** |
| **C** | **Measurement error** |  |  |  |  |  |  |  |  |  |  |  |  |  |

… Continued on the next page

| **D** | **Content validity** | **C13** | **C14** | **C15** | **C16** | **C17** |  | **C19** | **C20** | **C21** | **C22** | **C23** | **C24** | **C25** |
| --- | --- | --- | --- | --- | --- | --- | --- | --- | --- | --- | --- | --- | --- | --- |
| 1 | Assessment of whether all items refer to relevant aspects of the construct? | 0 | +++ | + | 0 | 0 |  | 0 | 0 | 0 | +++ | +++ | +++ | 0 |
| 2 | Assessment of whether all items are relevant for the study population | 0 | ++ | 0 | 0 | 0 |  | 0 | 0 | 0 | +++ | ++ | ++ | 0 |
| 3 | Assessment of whether all items are relevant for the purpose of instrument | + | +++ | + | + | + |  | ++ | + | + | +++ | ++ | + | + |
| 4 | Assessment of whether all items together reflect the construct? | + | +++ | + | 0 | + |  | +++ | 0 | + | 0 | 0 | 0 | + |
| 5 | Any important flaws in design or method of the study? | +++ | +++ | +++ | +++ | +++ |  | +++ | +++ | +++ | +++ | +++ | +++ | +++ |
|  | **Final score Box D** | **0** | **++** | **0** | **0** | **0** |  | **0** | **0** | **0** | **0** | **0** | **0** | **0** |
| **E** | **Structural validity** | **C13** | **C14** | **C15** | **C16** | **C17** |  |  | **C20** |  |  | **C23** | **C24** |  |
| 1 | Does scale consist of effect indicators, i.e. is it based on a reflective model? | ok | ok | ok | ok | ok |  |  | ok |  |  | ok | ok |  |
| 2 | Percentage of missing items given? | n/a | ++ | +++ | +++ | +++ |  |  | ++ |  |  | ++ | ++ |  |
| 3 | Description of how missing items were handled? | n/a | + | + | + | + |  |  | +++ |  |  | +++ | + |  |
| 4 | Sample size included in analysis adequate? | +++ | +++ | +++ | +++ | +++ |  |  | +++ |  |  | + | +++ |  |
| 5 | Any important flaws in design or method of the study? | +++ | +++ | +++ | +++ | +++ |  |  | +++ |  |  | + | +++ |  |
| 6 | For CTT: exploratory or confirmatory factor analysis performed? | +++ | +++ | +++ | +++ | +++ |  |  | +++ |  |  | +++ | +++ |  |
| 7 | For IRT: tests for determining (uni-) dimensionality of the items performed? | n/a | n/a | n/a | n/a | n/a |  |  | n/a |  |  | n/a | n/a |  |
|  | **Final score Box E** | **+++** | **+** | **+** | **+** | **+** |  |  | **++** |  |  | **+** | **+** |  |
| **F** | **Hypotheses testing** | **C13** |  | **C15** |  | **C17** | **C18** | **C19** | **C20** | **C21** |  | **C23** |  |  |
| 1 | Percentage of missing items given? | n/a |  | +++ |  | +++ | +++ | n/a | ++ | n/a |  | ++ |  |  |
| 2 | Description of how missing items were handled? | n/a |  | + |  | + | +++ | n/a | +++ | n/a |  | +++ |  |  |
| 3 | Sample size included in analysis adequate? | +++^p^/0^ph^ |  | +++ |  | +++ | +++ | 0 | + | + |  | 0 |  |  |
| 4 | Hypotheses regarding correlations or mean differences formulated a priori? | +++ |  | +++ |  | 0 | + | + | +++ | 0 |  | +++ |  |  |
| 5 | Expected direction of correlations or mean differences included in hypotheses? | +++ |  | +++ |  | ++ | +++ | ++ | +++ | ++ |  | +++ |  |  |
| 6 | Expected absolute/relative magnitude of correlations/mean differences included in hypotheses? | ++ |  | +++ |  | ++ | ++ | ++ | +++ | ++ |  | +++ |  |  |
| 7 | For convergent validity: adequate description provided of comparator instrument(s)? | +++ |  | n/a |  | + | +++ | n/a | +++ | n/a |  | +++ |  |  |
| 8 | For convergent validity: measurement properties of comparator instrument(s) adequately described? | +++ |  | n/a |  | 0 | +++ | n/a | 0 | n/a |  | +++ |  |  |
| 9 | Any important flaws in design or method of the study? | +++ |  | +++ |  | +++ | +++ | + | +++ | +++ |  | +++ |  |  |
| 10 | Design and statistical methods adequate for hypotheses to be tested? | +++ |  | +++ |  | +++ | +++ | 0 | ++ | +++ |  | +++ |  |  |
|  | **Final score Box F** | **++^P^/0^ph^** |  | **+** |  | **0** | **+** | **0** | **0** | **0** |  | **0** |  |  |
| **G** | **Cross-cultural validity** |  |  |  |  | **C17** |  |  |  |  |  |  | **C24** |  |
| 1 | Percentage of missing items given? |  |  |  |  | n/a |  |  |  |  |  |  | n/a |  |
| 2 | Description of how missing items were handled? |  |  |  |  | n/a |  |  |  |  |  |  | n/a |  |
| 3 | Sample size included in analysis adequate? |  |  |  |  | n/a |  |  |  |  |  |  | n/a |  |
| 4 | Both the original language in which instrument was developed and language in which instrument was translated described? |  |  |  |  | +++ |  |  |  |  |  |  | +++ |  |
| 5 | Expertise of people involved in translation process adequately described? |  |  |  |  | + |  |  |  |  |  |  | + |  |
| 6 | Did translators work independently from each other? |  |  |  |  | + |  |  |  |  |  |  | + |  |
| 7 | Items translated forward and backward? |  |  |  |  | 0 |  |  |  |  |  |  | + |  |
| 8 | Adequate description of how differences between the original and translated versions were resolved? |  |  |  |  | ++ |  |  |  |  |  |  | ++ |  |
| 9 | Translation reviewed by a committee? |  |  |  |  | ++ |  |  |  |  |  |  | ++ |  |
| 10 | Instrument pre-tested (e.g. cognitive interviews) to check interpretation, cultural relevance of the translation, and ease of comprehension? |  |  |  |  | 0 |  |  |  |  |  |  | +++ |  |
| 11 | Sample used in the pre-test adequately described? |  |  |  |  | n/a |  |  |  |  |  |  | + |  |
| 12 | Samples similar for all characteristics except language and/or cultural background? |  |  |  |  | n/a |  |  |  |  |  |  | n/a |  |
| 13 | Any important flaws in design or methods of the study? |  |  |  |  | n/a |  |  |  |  |  |  | n/a |  |
| 14 | For CTT: confirmatory factor analysis performed? |  |  |  |  | n/a |  |  |  |  |  |  | n/a |  |
| 15 | For IRT: differential item function (DIF) between language groups assessed? |  |  |  |  | n/a |  |  |  |  |  |  | n/a |  |
|  | **Final score Box G** |  |  |  |  | **0** |  |  |  |  |  |  | **+** |  |
| **H** | **Criterion validity** |  |  |  |  |  |  |  |  |  |  |  |  |  |
| **I** | **Responsiveness** |  |  |  |  |  |  |  |  |  |  |  |  |  |

* Description of item content altered to fit this table. For exact item content see COSMIN website (). Study IDs: C13 = Zandbelt et al., 2005; C14 = Makoul et al., 2007; C15 = Campbell et al., 2007; C16 = Baumann et al., 2008; C17 = Gulbrandsen et al., 2008; C18 = Fossli et al., 2011; C19 = Katsuyama et al., 2010; C20 = Bieber et al., 2010; C21 = Siminoff et al., 2011; C22 = McMilan et al., 2011; C23 = Salt et al., 2013; C24 = Wachira et al., 2013; C25 = Burt et al., 2014. 4-point scale rating: +++ = excellent, ++ = good, + = fair, 0 = poor, empty space = COSMIN rating not applicable. n/a = not applicable. Box B Reliability: a = Retest-Reliability, b = Interrater-Reliability, c = Intrarater-Reliability. If two reliability coefficients were calculated the rating refers to both, unless otherwise indicated. p=patient version, ph= physician version.
